# Daisaikoto for Menstrual Pain: A Lesson from a Case with Menstrual Pain Successfully Treated with Daisaikoto

**DOI:** 10.1155/2015/929514

**Published:** 2015-02-22

**Authors:** Yuko Horiba, Tetsuhiro Yoshino, Kenji Watanabe

**Affiliations:** Center for Kampo Medicine, Keio University School of Medicine, 35 Shinanomachi, Shinjuku, Tokyo 160-8582, Japan

## Abstract

Menstrual pain is one of the common symptoms among women. It is estimated that 5–14% of women are sometimes absent from school or work because of pain. Usually gynecologists prescribe analgesics and/or low-dose oral contraceptives. However, such treatment is not always effective and sometimes causes an adverse effect, such as stomach pain or low body temperature. Kampo medicine is one of the choices for the menstrual pain in Japan. Tokishakuyakusan, kamishoyosan, or keishibukuryogan is commonly used for the treatment of menstrual pain. Here we report a case of menstrual pain successfully treated with daisaikoto which is not commonly used for such a case. Twenty-five-year-old woman suffered from severe menstrual pain and stress at company. She also had constipation and abdominal distension. We prescribed daisaikoto extract 7.5 g per day. Not only menstrual pain but also constipation and abdominal distension improved within 6 months. Here we propose that daisaikoto is one of the choices for the treatment of menstrual pain with mental stress.

## 1. Introduction

Menstrual pain is one of the common symptoms among women. It is estimated that 5–14% of women are sometimes absent from school or work because of pain. The mechanism of menstrual pain is thought to be excessive uterine contraction and angiospasm due to prostaglandins F_2__*α*_ and E_2_. The very common treatment for the menstrual pain is the use of nonsteroidal anti-inflammatory drugs (NSAIDs) or low-dose oral contraceptives. NSAIDs, inhibitors of prostaglandin production, are useful treatment for approximately 60–70% of cases, but it makes only a temporary symptomatic relief of the pain and is harmful for the stomach mucosa causing gastritis or gastric ulcer. One of the options in such cases, for whom NSAIDs are not effective or are harmful, is Kampo treatment, Japanese traditional medicine. Most commonly used Kampo medicines for the treatment of menstrual pain are tokishakuyakusan, kamishoyosan, and keishibukuryogan, which are the 3 most frequently used Kampo formulas for women's health in general. However, traditional way to choose Kampo medicine is based on patient's pattern and not on a disease. We have reported 2 cases of menstrual pain that were successfully treated with daisaikoto [1]. Here, we report another case of menstrual pain that was successfully treated with daisaikoto and discuss the usefulness of daisaikoto for the treatment of menstrual pain.

## 2. Case Report

A 25-year-old female office worker visited the clinic of the Center for Kampo Medicine in Keio University Hospital. She suffered from severe menstrual pain since she was 20 years old and it turned to be worse when she started to work in the company. Also severe constipation started around that time. On the time of her visit, her bowel movement was once in 5 days. After she began to work, she was annoyed by acne and abdominal distension after meals. Physical examination revealed that she was 160 cm in height and 50 kg in weight; her body mass index was 19.5, blood pressure was 106/59 mmHg, and pulse was regular and at 63 per minute. Complexion and skin were normal. There were no abnormal findings on blood and urine analysis. Gynecological examination revealed no abnormal findings such as endometriosis. Tongue inspection revealed swelling of the sublingual vein. Abdominal strength was slightly strong; there were resistance in the hypochondrium and abdominal distention. We diagnosed the patient with excess heat, qi stagnation, and blood stasis pattern and prescribed 7.5 g of daisaikoto per day. Her constipation improved in 2 weeks. Twelve weeks later, she reported that her menstrual pain disappeared. Since then she has been free from a menstrual pain with daisaikoto.

## 3. Discussion

In this present case, menstrual pain was successfully treated with daisaikoto. We prescribed 2.5 g of daisaikoto extract (TJ-8; Tsumura Co., Tokyo, Japan) preprandially three times a day. One-day dose (7.5 g per day) contained 4.5 g of the compound extracts of 8 herbs: Bupleuri Radix (6 g), Pinelliae Tuber (4 g), Scutellariae Radix (3 g), Paeoniae Radix (3 g), Zizyphi Fructus (3 g), Aurantii Fructus Immaturus (2 g), Zingiberis Rhizoma (1 g), and Rhei Rhizoma (1 g). The extract product daisaikoto (TJ-8, Tsumura Daisaikoto Extract Granules) is a standardized spray-dried water extract, which includes magnesium stearate, lactose, and fructose fatty acid esters as diluents. The manufacturing process meets all requirements of the Japanese and international GMP guidelines. Usual indications of daisaikoto are hypertension, liver dysfunction, hyperlipidemia, nausea, vomiting, cholelithiasis, and diabetes [2].

Although daisaikoto is not a usual choice for menstrual pain, we have experienced previously two similar cases of menstrual pain improved with daisaikoto and have reported in Japanese [1]. Here we report an additional case and would like to discuss the possibility of daisaikoto for menstrual pain and clarify indications of daisaikoto for the relief of the menstrual pain, even though it is not widely thought to be as a first choice.

When we take these two cases and the present case together, there are several shared characteristics. First of all, all the patients had constipation. This is a very good target of daisaikoto, because daisaikoto contains Rhei Rhizoma which has a laxative effect. The second characteristic is a strong statue, which is called in Kampo medicine as an excess pattern which is listed in the traditional medicine chapter of the ICD-11 beta version [3].

In Kampo medical findings, abdominal strength is important and usually it is strong for daisaikoto [4]. The third and most important characteristic is the psychological stress. Two cases, which we reported before, had psychological stress at their school and home. The present case had psychological stress at her working environment. It has been reported that the psychological stress caused both menstrual cycle irregularities and menstrual pain among working women and college students [5].

Daisaikoto is known to treat the symptoms associated with psychological stress in the clinical settings even though the pharmacological mechanism has not been clarified [6]. Among 8 ingredients of daisaikoto, Bupleuri Radix and Aurantii Fructus Immaturus are described in the Japanese Pharmacopoeia to control the psychological stress [7].

In all these three cases, menstrual pain was improved within 6 months as well as stress associated symptoms, such as depressive mood, irritation, or headache. From these findings, the mechanism of daisaikoto for the menstrual pain was by controlling the psychological stress. All these three cases did not take any psychotropic drug or psychotherapy.

Taken together, we propose that when menstrual pain is associated with psychological stress, daisaikoto is one of the treatment options.

## 4. Conclusion

We report a case with menstrual pain successfully treated with daisaikoto. With two previously reported cases, we suggest that daisaikoto should be considered for the treatment of menstrual pain associated with psychological stress.
